# The Relationship Between Knowledge, Attitude, and Practice of Ergonomics and Musculoskeletal Disorders Amongst Dental Students

**DOI:** 10.1155/ijod/5203500

**Published:** 2026-09-25

**Authors:** Doha Beshir, Dareen Kiblawi, Nour Eyad, Fawaz Pullishery, Divya Gopinath

**Affiliations:** ^1^ College of Dentistry, Ajman University, Al Jurf, Ajman, UAE, ajman.ac.ae; ^2^ Division of Dental Public Health, Faculty of Dentistry, Batterjee Medical College, Jeddah, Saudi Arabia, bmc.edu.sa; ^3^ Basic Medical and Dental Sciences Dept, College of Dentistry, Ajman University, Al Jurf, Ajman, UAE, ajman.ac.ae

**Keywords:** dental education, dental students, ergonomics, musculoskeletal disorders, occupational health

## Abstract

**Background:**

Musculoskeletal disorders (MSDs) are among the most common occupational health problems affecting dental professionals and students. However, evidence linking ergonomic knowledge, attitude, and practices with MSDs remains limited.

**Materials and Methods:**

A cross‐sectional, questionnaire‐based study was conducted among clinical undergraduate dental students, interns, and postgraduate trainees at a tertiary academic institution in the United Arab Emirates. A total of 240 participants completed a validated online survey assessing sociodemographic characteristics, work‐related factors, ergonomic knowledge, attitudes, practices, and musculoskeletal health status. Associations between participant characteristics, ergonomic factors, and MSDs were evaluated using Chi‐square tests and multivariable logistic regression analyses.

**Results:**

Overall, participants demonstrated a high level of awareness of general ergonomics and a moderate level of practice. A significant association was observed between working ≥6 h per day and ergonomic knowledge (*p* = 0.019). Similarly the use of magnification devices was significantly associated with higher ergonomic knowledge (*p* = 0.028) and better ergonomic practices (*p* < 0.001) compared with non use. The overall prevalence of MSDs among the participants was 75.0%; prevalence was significantly higher among participants aged 25 years or older, females, and those in more advanced stages of training (*p* < 0.05). Multivariable logistic regression analysis further identified age ≥25 years (OR = 7.45), female gender (OR = 2.91), poor ergonomic knowledge (OR = 11.97), and poor ergonomic practices (OR =10.66) as significant independent predictors of MSDs.

**Conclusion:**

Although most participants demonstrated good ergonomic knowledge and positive attitudes, inconsistent ergonomic practices remained common, contributing to a high prevalence of MSDs. These findings highlight the need to strengthen practical ergonomic training and preventive measures within dental education.

## 1. Introduction

Ergonomics can be defined as “an applied science concerned with designing and arranging things people use so that people and things interact most efficiently and safely” [1]. The association between “labor” and “health,” indicating health problems acquired from work, has been documented since ancient times, including in ancient Egypt and the Greek and Roman periods [2]. Ergonomics aims to align equipment and work set ups to reduce biomechanical strain on professionals [3]. In dentistry, practicing dental ergonomics can improve work quality and enhance productivity [4]. By applying ergonomic principles, dental professionals can perform clinical procedures more comfortably while minimizing physical stress on their bodies. This helps maintain efficiency during treatment and allows clinicians to work for longer periods without excessive fatigue.

Dental practice demands precise skill due to the confined working space, mostly within the oral cavity, where specific and recurrent force is required to deliver dental care. Dentists often need to maintain close visual focus and fine motor control while performing procedures. This frequently requires bending the neck, rotating the back, and elevating the shoulders for prolonged periods. Such repetitive movements and awkward postures place continuous strain on the musculoskeletal system. In addition, dental procedures are often lengthy and require high levels of concentration, which further contributes to physical tension. These working conditions lead to maintaining a fixed posture for extended periods, making dentists susceptible to occupational hazards [4, 5]. As a result, dentists are often prone to multiple undesirable health conditions, with musculoskeletal disorders (MSDs) being the most commonly reported among the occupation [6].

MSDs represent a major occupational hazard for dental professionals. They account for 11%–98% of occupational health issues, with an onset as early as dental school [7]. Dental students are frequently exposed to the same risk factors as those of practicing dentists, including prolonged sitting, repetitive hand movements, and improper posture during clinical and laboratory sessions. At this stage of training, students are primarily focused on developing their technical skills and completing clinical requirements, sometimes overlooking the importance of maintaining proper ergonomics. As a result, poor working habits may develop early and continue throughout their professional careers if not corrected [8]. According to a meta‐analysis from Western nations, the most commonly affected areas in dental professionals include the neck (58.5%), lower back (56.4%), shoulders (43.1%), and upper back (41.1%) [9]. Continuous strain on these areas can gradually lead to discomfort, stiffness, and pain during or after clinical work. Their effects extend beyond tolerable discomfort, often leading to lower job satisfaction, limited work potential, and possible job‐related accidents, thus resulting in temporary leave and early retirement [9, 10]. In severe cases, chronic MSDs may even force dental professionals to reduce their working hours or discontinue clinical practice entirely. Therefore, prevention and early awareness of these conditions are essential for maintaining long‐term occupational health within the dental profession.

Proper ergonomics is crucial for ensuring safety and maintaining work efficiency, thereby facilitating superior clinical treatment [11]. Interventions to prevent MSDs amongst dental professionals, such as changing individual behavior, have been evaluated [12]. Correct posture, appropriate patient positioning, and the use of ergonomically designed instruments can significantly reduce physical strain during dental procedures. In addition, taking regular breaks and performing stretching exercises may help relieve muscle tension and prevent fatigue. When ergonomic principles are properly applied, dentists can maintain a better posture and reduce the risk of musculoskeletal problems. However, the scope of ergonomics in dentistry is vast, making it challenging to fully abide by. The clinical environment, patient positioning, equipment design, and operator habits all play a role in determining whether ergonomic principles are successfully implemented [13]. Although the knowledge of ergonomics is familiar amongst dental professionals, it is not regularly practiced. Hence, signifying the importance of assessing not only knowledge but also attitude and practice regarding ergonomics [13–16]. Despite its major influence on a dentist’s well‐being, there are still insufficient awareness and studies on the topic, especially among dental students [1, 17–20]. Although numerous studies have reported the prevalence of work‐related MSDs among dental students and professionals, relatively few have comprehensively examined the relationship between ergonomic knowledge, attitudes, practices, and MSDs. Consequently, the extent to which ergonomic awareness and behaviors independently contribute to the occurrence of MSDs remains incompletely understood. Therefore, this study intends to further investigate the relationship between ergonomic knowledge, attitudes, and practices and MSDs.

## 2. Materials and Methods

### 2.1. Study Design and Participants

This cross‐sectional study was conducted between November 2025 and March 2026 at Ajman University, United Arab Emirates (UAE). The study targeted undergraduate clinical dental students, dental interns, and postgraduate dental trainees who were actively involved in clinical dental procedures. Undergraduate students in Year 1 and Year 2 were excluded because they were not routinely engaged in extensive clinical dental practice or patient management activities. Participation in the study was voluntary, and only individuals who provided informed consent were included.

The study protocol was reviewed and approved by the Ajman University Research Ethics Committee under project number D‐F‐H‐31‐Oct‐2025. All procedures were conducted in accordance with the ethical principles outlined by the institutional research committee and the Declaration of Helsinki. Participants were informed about the study’s objectives, the confidentiality of the collected data, the voluntary nature of participation, and their right to withdraw at any stage without penalty. No personal identifiers were collected, and all responses were recorded anonymously.

### 2.2. Sample Size Calculation

The minimum required sample size was calculated for this cross‐sectional study using a 95% confidence level, a 5% margin of error, and an anticipated prevalence of 50%, which was selected to provide the most conservative estimate in the absence of prior prevalence data. Given an accessible population of approximately 600 eligible clinical undergraduate dental students, dental interns, and postgraduate trainees in the participating institutions, a finite population correction was applied. The minimum required sample size was estimated to be 235 participants. A total of 240 complete responses were obtained and included in the final analysis, exceeding the calculated minimum sample size.

### 2.3. Data Collection

**Figure 1:**
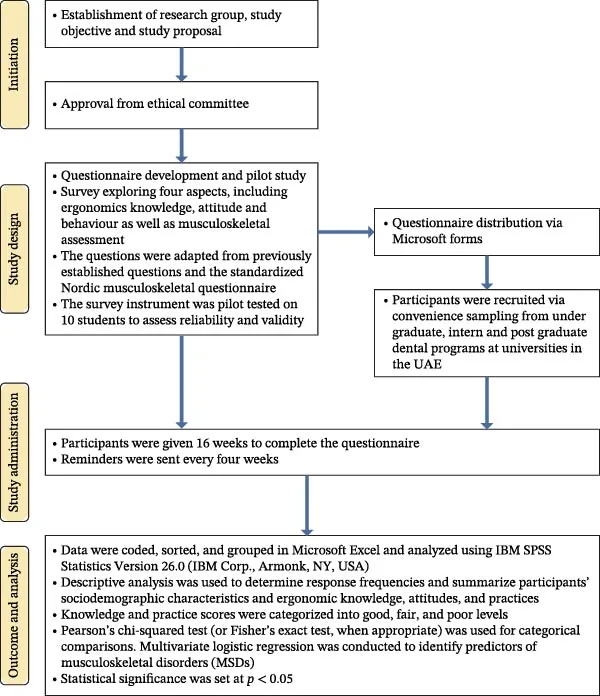
An overview of the study design and survey process.

Data were collected using a self‐administered, anonymous online questionnaire developed through Microsoft Forms. An overview of the study design and survey process is presented in Figure 1. The survey link was distributed electronically through institutional communication channels and student social media platforms commonly used within dental colleges in the UAE. The questionnaire was administered in English, and translation was not considered necessary because dental education in the UAE is primarily conducted in English.

The online survey invitation included detailed information on the study’s purpose, the confidentiality of responses, the estimated completion time, and an informed consent statement. Participants were required to confirm their consent electronically before proceeding with the questionnaire. The survey remained open for 16 weeks, and reminder notifications were sent every 4 weeks to improve the response rate.

The questionnaire consisted of three sections. The first section included six questions assessing sociodemographic and work‐related characteristics, such as age, gender, educational level, clinical working hours, assistant availability, and use of magnification devices, as all of these factors were considered potentially relevant. The second section consisted of 18 questions evaluating knowledge, attitudes, and ergonomic practices related to dental ergonomics. These questions were adapted from a previously validated questionnaire, which demonstrated good reliability. The reported Cronbach’s alpha and intraclass correlation coefficient (ICC) values indicated good reliability for the knowledge (*α* = 0.931; ICC = 0.919), attitude (*α* = 0.840; ICC = 0.829), and practice (*α* = 0.737; ICC = 0.876) domains. All items showed corrected item‐total correlation (CITC) values of > 0.3, confirming acceptable internal consistency [18].

The third section included the standardized Nordic musculoskeletal questionnaire (NMQ), which assessed the presence and distribution of musculoskeletal symptoms in different body regions during clinical practice [15]. The instrument evaluates musculoskeletal symptoms across nine anatomical regions: the neck, shoulders, elbows, wrists/hands, upper back, lower back, hips/thighs, knees, and ankles/feet. Participants were asked to indicate whether they had experienced pain, discomfort, or numbness in each anatomical region during the specified recall period and whether these symptoms had interfered with their daily activities or clinical work.

For each anatomical region, responses were recorded dichotomously as “yes” or “no.” The prevalence of symptoms was calculated as the proportion of participants reporting symptoms in each region. For the overall MSD variable, participants who reported symptoms in at least one anatomical region were classified as having an MSD, whereas those reporting no symptoms in any region were classified as not having an MSD. No changes were made to the anatomical regions or the underlying meaning of the original items.

A pilot study was conducted with 10 dental students to evaluate the questionnaire’s clarity, relevance, feasibility, and internal consistency. Informal verbal feedback obtained from the pilot participants confirmed that the questions were understandable, relevant, and appropriately structured. Therefore, no modifications were required before the administration of the main survey. To minimize potential bias, participants included in the pilot study were excluded from the final study sample.

### 2.4. Statistical Analysis

Descriptive statistics were initially conducted to outline the sociodemographic characteristics of participants and their ergonomic knowledge, attitudes, and practices. Associations between ergonomic knowledge, sociodemographic characteristics, ergonomic‐related variables, and MSDs were assessed using the Chi‐square test. Knowledge and practice scores were calculated by assigning one point to each correct (knowledge) or ergonomically appropriate (practice) response and zero points to incorrect or inappropriate responses. Individual scores were summed to obtain total knowledge and practice scores for each participant. To facilitate comparative analysis, participants were categorized into three levels based on the percentage of the maximum attainable score: good (≥75%), fair (50%–74%), and poor (<50%). These categories were subsequently used in association and regression analyses. Variables showing significant associations in univariable analysis were included in the multivariable regression model to identify independent predictors of MSDs. Adjusted odds ratios (AORs) with 95% confidence intervals (CIs) were reported. To identify independent predictors of MSD, multivariable logistic regression analysis was performed. Prior to multivariable logistic regression, multicollinearity among independent variables was assessed using variance inflation factors (VIFs) and tolerance values. VIF values <5 and tolerance values >0.2 were considered indicative of the absence of problematic multicollinearity. Variables with a *p*‐value <0.20 in bivariate analysis, along with clinically relevant variables, were included in the regression model. AORs with 95% CIs were reported to quantify the strength of associations. All statistical tests were two‐tailed, and statistical significance was set at *p*  < 0.05. The data were analyzed using IBM SPSS Statistics (Version 26.0, IBM Corp., Armonk, NY, USA).

## 3. Results

### 3.1. Participant Characteristics

The online questionnaire was distributed to approximately 600 eligible clinical undergraduate dental students, dental interns, and postgraduate dental trainees across the participating institutions. A total of 240 completed questionnaires were returned, yielding a response rate of 40.0%, and all completed questionnaires were included in the final analysis. The majority of participants were aged under 25 years (73.8%), while females accounted for 62.5% of the sample. Participants were distributed across undergraduate clinical years, internship, and postgraduate levels. Most participants reported working 6 h or more daily (69.6%), and 65.0% had access to a dental assistant. However, the use of magnification devices was relatively low, with only 26.7% reporting regular use. The sociodemographic characteristics and selected ergonomic‐related variables of the study participants are presented in Table 1.

**Table 1:** Sociodemographic characteristics and ergonomic knowledge of participants (*N* = 240).

Variable	Category	Frequency (*n*)	Percentage (%)
Age	<25 Years	177	73.8
	≥25 Years	63	26.3
Gender	Female	150	62.5
	Male	90	37.5
Year of education	3^rd^ Year	45	18.8
	4^th^ Year	42	17.5
	5^th^ Year	52	21.7
	Internship	52	21.7
	Postgraduate	49	20.4
Working hours/day	<6 h	73	30.4
	≥6 h	167	69.6
Assistant availability	No	84	35.0
	Yes	156	65.0
Use of magnification	No	176	73.3
	Yes	64	26.7

### 3.2. Ergonomic Knowledge, Attitudes, and Practices

Overall, participants demonstrated a high level of awareness of general ergonomic concepts and the health risks associated with poor ergonomics. Full knowledge of ergonomics was reported by 94.6% and awareness of health hazards related to poor ergonomics by 88.8%. Knowledge of sitting posture and harmful postures was relatively high, whereas awareness of correct shoulder, elbow, forearm, and finger positioning was comparatively low. A detailed breakdown of participants’ responses to ergonomics knowledge questions is presented in Tables S1 and S2. Most participants also expressed positive attitudes toward incorporating ergonomics into the dental curriculum and continuing education programs (Table S3). Despite good knowledge and attitudes, the ergonomic practices were only moderate. Most participants reported maintaining neutral posture and proper sitting positions “often” rather than “always.” The use of dental loupes was notably limited, with 43.8% reporting never using them. Ergonomic practice patterns are presented in Table S4.

### 3.3. Factors Associated With Ergonomic Knowledge and Practice

The association between participant characteristics and ergonomic knowledge is shown in Table 2. Participants working more than 6 h per day demonstrated significantly better ergonomic knowledge compared with those working fewer than 6 h (*p* = 0.019). Similarly, participants who used magnification devices showed higher levels of ergonomic knowledge (*p* = 0.028) (Table 2) and better ergonomic practices (*p* < 0.001) than nonuser (Table 3). No significant association was observed with age, gender, year of education, or assistant availability (*p*  > 0.05).

**Table 2:** Association between participant characteristics and self‐reported knowledge of ergonomics.

Variable	Category	Self‐reported knowledge			*p*‐Value
		Good	Fair	Poor	
Age	<25 Years	71 (40.1)	84 (47.5)	22 (12.4)	0.622
	≥25 Years	27 (42.9)	31 (49.2)	5 (7.9)	
Gender	Female	56 (37.3)	79 (52.7)	15 (10.0)	0.162
	Male	42 (46.7)	36 (40.0)	12 (13.3)	
Year of education	3^rd^ Year	16 (35.6)	23 (51.1)	6 (13.3)	0.168
	4^th^ Year	10 (23.8)	29 (69.0)	3 (7.1)	
	5^th^ Year	26 (50.0)	21 (40.4)	5 (9.6)	
	Internship	23 (44.2)	22 (42.3)	7 (13.5)	
	Postgraduate	23 (46.9)	20 (40.8)	6 (12.2)	
Working hours/day	<6 h	31 (42.5)	28 (38.4)	14 (19.2)	0.019
	≥6 h	67 (40.1)	87 (52.1)	13 (7.8)	
Assistant availability	No	31 (36.9)	40 (47.6)	13 (15.5)	0.280
	Yes	67 (42.9)	75 (48.1)	14 (9.0)	
Use of magnification	No	64 (36.4)	88 (50.0)	24 (13.6)	0.028
	Yes	34 (53.1)	27 (42.2)	3 (4.7)	

**Table 3:** Association between participant characteristics and ergonomic practices.

Variable	Category	Ergonomic practices			*p*‐Value
		Good	Fair	Poor	
Age	<25 Years	125 (70.6)	32 (18.1)	20 (11.3)	0.601
	≥25 Years	42 (66.7)	15 (23.8)	6 (9.5)	
Gender	Female	98 (65.3)	35 (23.3)	17 (11.3)	0.135
	Male	69 (76.7)	12 (13.3)	9 (10.0)	
Year of education	3^rd^ Year	27 (60.0)	14 (31.1)	4 (8.9)	0.134
	4^th^ Year	28 (66.7)	6 (14.3)	8 (19.0)	
	5^th^ Year	42 (80.8)	6 (11.5)	4 (7.7)	
	Internship	37 (71.2)	12 (23.1)	3 (5.8)	
	Postgraduate	33 (67.3)	9 (18.4)	7 (14.3)	
Working hours/day	<6 h	47 (64.4)	18 (24.7)	8 (11.0)	0.406
	≥6 h	120 (71.9)	29 (17.4)	18 (10.8)	
Assistant availability	No	54 (64.3)	21 (25.0)	9 (10.7)	0.292
	Yes	113 (72.4)	26 (16.7)	17 (10.9)	
Use of magnification	No	110 (62.5)	41 (23.3)	25 (14.2)	<0.001
	Yes	57 (89.1)	6 (9.4)	1 (1.6)	

A statistically significant association was observed between self‐reported knowledge and ergonomic practices among participants. Participants with good knowledge demonstrated the highest proportion of good ergonomic practices (81.6%), while those with fair knowledge showed a moderate level of good practices (65.2%), as shown in Table 4 (*p* = 0.002). Also, participants with poor knowledge had the lowest proportion of good practices (44.4%) and the highest proportion of poor practices (25.9%).

**Table 4:** Association between self‐reported knowledge and ergonomic practices.

Self‐reported knowledge	Ergonomic practices			*p*‐Value
	Good	Fair	Poor	
Good	80 (81.6)	13 (13.3)	5 (5.1)	0.002
Fair	75 (65.2)	26 (22.6)	14 (12.2)	
Poor	12 (44.4)	8 (29.6)	7 (25.9)	

### 3.4. Prevalence and Predictors of MSDs

The overall prevalence of MSDs among the participants was 75.0%, with 180 out of 240 individuals reporting at least one musculoskeletal complaint in the past 12 months. Lower back symptoms (54.2%) and neck symptoms (51.2%) were the most commonly reported musculoskeletal complaints and were associated with the greatest impact on daily activities and clinical work. Shoulders (37.9%), wrists/hands (35.0%), and upper back (33.3%) were also frequently affected. The overall prevalence and distribution of musculoskeletal symptoms are summarized in Tables S5 and S6, respectively.

Age showed astrong association: participants aged ≥25 years had a markedly higher prevalence of MSD (96.8%) than those <25 years (67.2%); *p*  < 0.001. Gender was also significantly associated, with females reporting a higher prevalence (80.7%) than males (65.6%) (*p* = 0.009). A significant trend was observed across the years of education (*p* = 0.001), with MSD prevalence increasing progressively from undergraduate to postgraduate levels, reaching as high as 98.0% among postgraduates. At the same time, working hours per day, assistant availability, and use of magnification were not significantly associated with MSD prevalence (*p*  > 0.05), although higher prevalence was noted among those working fewer hours and those using magnification. Importantly, self‐reported ergonomic knowledge and practices were significantly associated with MSD. Participants with poor knowledge (96.3%) and poor practices (96.2%) had substantially higher prevalence compared to those with good knowledge (67.3%) and good practices (67.7%) (*p*  < 0.05). Associations between participant characteristics and MSDs are presented in Table 5.

**Table 5:** Association between participant characteristics and prevalence of musculoskeletal disorders.

Variable	Category	Prevalence		*p*‐Value
		No	Yes	
Age	<25 Years	58 (32.8)	119 (67.2)	<0.001
	≥25 Years	2 (3.2)	61 (96.8)	
Gender	Female	29 (19.3)	121 (80.7)	0.009
	Male	31 (34.4)	59 (65.6)	
Year of education	3^rd^ Year	15 (33.3)	30 (66.7)	0.001
	4^th^ Year	15 (35.7)	27 (64.3)	
	5^th^ Year	17 (32.7)	35 (67.3)	
	Internship	12 (23.1)	40 (76.9)	
	Postgraduate	1 (2.0)	48 (98.0)	
Working hours/day	<6 h	15 (20.5)	58 (79.5)	0.292
	≥6 h	45 (26.9)	122 (73.1)	
Assistant availability regularly	No	17 (20.2)	67 (79.8)	0.221
	Yes	43 (27.6)	113 (72.4)	
Use of magnification	No	49 (27.8)	127 (72.2)	0.092
	Yes	11 (17.2)	53 (82.8)	
Self‐reported knowledge	Good	32 (32.7)	66 (67.3)	0.008
	Fair	27 (23.5)	88 (76.5)	
	Poor	1 (3.7)	26 (96.3)	
Practices	Good	54 (32.3)	113 (67.7)	<0.001
	Fair	5 (10.6)	42 (89.4)	
	Poor	1 (3.8)	25 (96.2)	

A multivariable logistic regression analysis was done to identify predictors of MSD among participants. Age was a significant predictor, with individuals aged ≥25 years demonstrating 7.45 times higher odds of developing MSD compared to younger participants (*p* = 0.015) (Table 6 and Table S7). Gender also showed a significant association, where females had 2.91 times higher odds of MSD compared to males (*p* = 0.003). Among the ergonomic factors, both poor ergonomic practices and poor ergonomic knowledge emerged as strong independent predictors. Participants with poor practices had 10.66 times higher odds of MSD (*p* = 0.028), while those with poor knowledge had 11.97 times higher odds (*p* = 0.021), indicating a substantial impact of ergonomics on musculoskeletal health. But variables such as year of education, working hours, assistant availability, and use of magnification were not statistically significant predictors (*p*  > 0.05), although some showed trends toward increased or decreased risk.

**Table 6:** Multivariable logistic regression analysis of factors associated with musculoskeletal disorders (MSD).

Variable	Category	Adjusted OR (Exp[B])	95% CI	*p*‐Value
Age	≥25 Years	7.45	1.47–37.64	0.015 ^∗^
	25 Years (ref)	—	—	—
Gender	Female	2.91	1.45–5.98	0.003 ^∗^
	Male (ref)	—	—	—
Year of education	3^rd^ (Ref)	—	—	—
	4^th^	7.23	0.58–89.91	0.124
	5^th^	5.71	0.53–61.40	0.150
	Internship	5.90	0.58–60.59	0.135
	Postgraduate	4.08	0.38–44.32	0.248
Working hours/day	≥6 h	0.78	0.30–2.01	0.605
	<6 h (Ref)	—	—	—
Assistant availability	Yes	0.54	0.12–2.36	0.409
	No (ref)	—	—	—
Use of magnification	Yes	2.06	0.83–5.12	0.118
	No (ref)	—	—	—
Ergonomic practices	Poor	10.66	1.30–87.81	0.028 ^∗^
Ergonomic knowledge	Poor	11.97	1.45–98.58	0.021 ^∗^

*Note*: ( ^∗^) statistically significant at *p* < 0.05.

## 4. Discussion

The findings of the current study demonstrated high levels of ergonomic awareness and positive attitudes toward ergonomics. Most participants reported familiarity with ergonomics and recognized its importance in preventing occupational health problems. In addition, the majority strongly supported the inclusion of ergonomics within the dental curriculum and continuing dental education programs. These findings are consistent with previous international studies conducted in Indonesia, Brazil, Poland, and Morocco, where dental students and practitioners also demonstrated positive attitudes toward ergonomics [4, 18, 21, 22]. However, despite adequate awareness, practical implementation remained inconsistent. Many participants reported following ergonomic principles “often” rather than “always,” suggesting a gap between theoretical knowledge and actual clinical behavior. These findings indicate that awareness alone may not be sufficient to ensure appropriate ergonomic behavior during clinical practice. Similar findings have been reported in previous studies, in which dental students understood ergonomic principles but had difficulty applying them consistently during patient care [4, 21]. Nurses, dentists, and students have reported that time constraints, lack of space, and demanding heavy workload make it hard to maintain ideal posture or use equipment correctly [23–25]. Dental students and trainees are frequently exposed to prolonged static postures, repetitive hand movements, awkward neck flexion, and visually demanding procedures [26]. Under such conditions, maintaining an ideal posture throughout clinical procedures may become difficult despite adequate ergonomic awareness. Moreover, clinical training often prioritizes procedural competency and treatment completion, while ergonomic posture correction may receive comparatively less emphasis during supervision. This may contribute to the persistence of poor ergonomic habits, even among knowledgeable individuals. Thus, additional training in dental ergonomics is required to reduce work‐related complications. This finding is clinically important because improper upper limb positioning contributes significantly to muscle fatigue, repetitive strain injuries, and chronic MSD development [27–29]. Therefore, ergonomic education should not focus solely on theoretical concepts but should include repeated practical reinforcement through simulation sessions, clinical observation, posture monitoring, and direct feedback during clinical procedures.

The present study found no statistically significant association between age and ergonomic knowledge, which is consistent with findings reported by Kalghatgi et al. [30] and El‐sallamy et al. [31]. However, Galla et al. [20] reported contrary findings, with younger dentists demonstrating poorer ergonomic knowledge than older practitioners. Similarly, gender did not demonstrate a statistically significant association with ergonomic knowledge in the present study, which aligns with studies by Galla et al. [20] and El‐sallamy et al. [31]. In contrast, another study from Saudi Arabia reported significant gender‐related differences in ergonomic awareness and practice [32]. The educational level also did not significantly influence ergonomic knowledge in the current study, consistent with findings reported by Febriana Setiawati et al. [18]. However, several previous studies have observed that increased expertise and higher educational training significantly improve ergonomic awareness and practices [32–34]. The present study also identified low utilization of magnification devices, with many participants reporting no use of dental loupes. Previous studies have demonstrated that magnification systems improve posture, reduce neck flexion, and decrease musculoskeletal strain during dental procedures [35, 36]. In the current study, participants who used magnification devices demonstrated significantly better ergonomic knowledge than nonusers. This may indicate that individuals using ergonomic aids are generally more conscious of occupational health risks and preventive ergonomic practices. Working more than 6 h daily was significantly associated with better ergonomic knowledge. Greater clinical exposure may increase awareness regarding occupational strain and encourage the acquisition of ergonomic knowledge. Similar findings have been reported previously, where increased clinical experience contributed to improved ergonomic awareness and expertise [33, 34]. However, prolonged clinical hours may simultaneously increase cumulative musculoskeletal loading, emphasizing the importance of early preventive ergonomic interventions.

A major finding of this study was the high prevalence of MSDs among participants. Overall, 75.0% reported MSD‐related symptoms during the previous 12 months. The lower back and neck were the most commonly affected regions, followed by the shoulders, wrists/hands, and upper back. Lower back pain demonstrated the greatest impact on daily activities and work performance. These findings are consistent with studies conducted in Saudi Arabia and Germany [37, 38], as well as the meta‐analysis by Almeida et al. [8], which identified the neck, lower back, and shoulders as the most commonly affected anatomical regions among dental students and practitioners. The high prevalence of lower back and neck symptoms is likely related to the postural demands of dental practice. Dentistry commonly requires prolonged forward head posture, trunk flexion, elevated shoulders, and static seated positions for extended periods. Over time, these repetitive biomechanical stresses contribute to muscle imbalance, fatigue, and chronic musculoskeletal strain. Therefore, MSDs remain a major occupational health concern within the dental profession globally [37, 38].

Several participant‐related factors were significantly associated with MSD prevalence. Participants aged ≥25 years demonstrated significantly greater MSD prevalence than that of younger participants, while postgraduate trainees reported the highest prevalence among educational levels. These findings are consistent with previous studies reporting a higher prevalence of MSDs among older participants and those at more advanced stages of dental training, although the underlying reasons are likely multifactorial [37]. Female participants demonstrated significantly greater MSD prevalence than males, consistent with previous studies and systematic reviews [37, 38]. Potential explanations may include differences in muscle endurance, posture adaptation, workload tolerance, and psychosocial responses. However, gender‐related MSD variation remains multifactorial and may differ across populations and workplace environments [30, 31]. Importantly, poor ergonomic knowledge and poor ergonomic practices were strongly associated with MSD prevalence. Participants with inadequate ergonomic knowledge and practices exhibited markedly higher musculoskeletal symptoms than those with good ergonomic behavior. Multivariable logistic regression analysis further identified poor ergonomic knowledge and poor ergonomic practices as independent predictors of MSDs. These findings support previous evidence identifying poor ergonomic knowledge and practices as important risk factors for MSD development among dental students and professionals [8, 26, 39].

The findings of this study have important implications for dental education and occupational health prevention. Ergonomic principles should be thoroughly integrated early within undergraduate dental curricula and reinforced continuously throughout clinical training. Educational interventions can include practical ergonomic workshops, posture evaluation, simulation‐based ergonomic training, magnification use training, and supervised chairside correction. In addition, promoting regular stretching exercises, physical activity, and scheduled rest breaks may help reduce cumulative musculoskeletal strain during clinical work [8, 13, 39].

This study has several strengths. It evaluated ergonomics comprehensively by assessing knowledge, attitudes, practices, and musculoskeletal outcomes simultaneously among participants across multiple educational levels. The study also identified modifiable ergonomic factors associated with MSDs, providing clinically relevant information that may guide preventive educational strategies. However, several limitations should be acknowledged. The cross‐sectional study design limits the ability to establish the causality between ergonomic factors and MSDs. In addition, data were collected via self‐report questionnaires, which may introduce recall and social desirability biases. Ergonomic practices were also evaluated through self‐reporting rather than through direct clinical observation. Finally, the study population was limited to dental trainees in one institution in UAE, which may reduce generalizability.

## 5. Conclusion

MSDs were highly prevalent among dental students, interns, and postgraduate trainees, with the lower back and neck being the most commonly affected regions. Poor ergonomic knowledge and inconsistent ergonomic practices were significant independent predictors of MSDs alongside age and gender. Although most participants demonstrated positive attitudes toward ergonomics, gaps in practical implementation remained evident. These findings emphasize the need for early integration of ergonomic education, practical training, and preventive strategies within dental curricula to reduce the burden of MSDs and promote long‐term occupational health among future dental professionals.

## Funding

The APC was funded by the Ajman University.

## Disclosure

The authors take full responsibility for the content of the manuscript.

## Ethics Statement

This study was approved by the Ajman University Research Ethics Committee under Project Number D‐F‐H‐31‐Oct‐2025.

## Conflicts of Interest

The authors declare no conflicts of interest.

## Supporting Information

Additional supporting information can be found online in the Supporting Information section.

## Supporting information


**Supporting Information** Table S1: Knowledge of ergonomics among participants. Table S2: Advanced ergonomic knowledge parameters. Table S3: Attitudes toward ergonomics in dentistry. Table S4: Ergonomic practice patterns. Table S5: Overall prevalence of musculoskeletal disorders (MSD). Table S6: Prevalence, impact, healthcare‐seeking, and work limitation of musculoskeletal symptoms. Table S7: Logistic Regression Model Diagnostics.

## Data Availability

The data that support the findings of this study are available from the corresponding author upon reasonable request.
