# Supplementary material for: The Relationship Between Knowledge, Attitude, and Practice of Ergonomics and Musculoskeletal Disorders Amongst Dental Students
Source: Int J Dent. 2026 Sep 25;2026:5203500. doi: 10.1155/ijod/5203500 (PMC13613095; doi:10.1155/ijod/5203500)
Supplement: Supplementary file 1 [file IJOD-2026-5203500-s001.docx]

| **ST1: Knowledge of Ergonomics Among Participants** | | | |
| --- | --- | --- | --- |
| **Variable** | **Category** | **Frequency (n)** | **Percentage (%)** |
| **Know Ergonomics** | No | 5 | 2.1 |
|  | To some extent | 8 | 3.3 |
|  | Yes | 227 | 94.6 |
| **Know Health Hazards** | No | 9 | 3.8 |
|  | To some extent | 18 | 7.5 |
|  | Yes | 213 | 88.8 |
| **Know Benefits of Ergonomics** | No | 8 | 3.3 |
|  | To some extent | 14 | 5.8 |
|  | Yes | 218 | 90.8 |
| **Know Harmful Posture** | No | 17 | 7.1 |
|  | To some extent | 72 | 30.0 |
|  | Yes | 151 | 62.9 |
| **Know Best Sitting Posture** | No | 9 | 3.8 |
|  | To some extent | 46 | 19.2 |
|  | Yes | 185 | 77.1 |
| **Know Shoulder/Elbow Position** | No | 19 | 7.9 |
|  | To some extent | 82 | 34.2 |
|  | Yes | 139 | 57.9 |
| **Know Forearm & Finger Position** | No | 29 | 12.1 |
|  | To some extent | 101 | 42.1 |
|  | Yes | 110 | 45.8 |

**Supplementary Table 1: Knowledge of Ergonomics Among Participants**

| **ST2: Advanced Ergonomic Knowledge Parameters** | | | |
| --- | --- | --- | --- |
| **Variable** | **Category** | **Frequency (n)** | **Percentage (%)** |
| **Sight-line & Light-line** | No | 50 | 20.8 |
|  | To some extent | 107 | 44.6 |
|  | Yes | 83 | 34.6 |
| **Contact Points (Stability)** | No | 46 | 19.2 |
|  | To some extent | 110 | 45.8 |
|  | Yes | 84 | 35.0 |
| **Treatment Room Design** | No | 41 | 17.1 |
|  | To some extent | 108 | 45.0 |
|  | Yes | 91 | 37.9 |
| **Body Motion & Instrument Support** | No | 48 | 20.0 |
|  | To some extent | 117 | 48.8 |
|  | Yes | 75 | 31.3 |
| **Orbit Around Patient’s Head** | No | 61 | 25.4 |
|  | To some extent | 113 | 47.1 |
|  | Yes | 66 | 27.5 |
| **Ergonomic Headrest** | No | 32 | 13.3 |
|  | To some extent | 74 | 30.8 |
|  | Yes | 134 | 55.8 |
| **Ideal Working Distance** | No | 51 | 21.3 |
|  | To some extent | 110 | 45.8 |
|  | Yes | 79 | 32.9 |
| **Exercise Between Patients** | No | 67 | 27.9 |
|  | To some extent | 107 | 44.6 |
|  | Yes | 66 | 27.5 |
| **Maintain Environment (Light/Temp)** | No | 32 | 13.3 |
|  | To some extent | 114 | 47.5 |
|  | Yes | 94 | 39.2 |

S**upplementary Table 2: Advanced Ergonomic Knowledge Parameters**

| **ST3: Attitudes Toward Ergonomics in Dentistry (N = 240)** | | | | | |
| --- | --- | --- | --- | --- | --- |
| **Variable** | **Definitely No** | **No** | **Neutral** | **Yes** | **Definitely Yes** |
| Ergonomics in Curriculum | 2 (0.8) | 1 (0.4) | 12 (5.0) | 50 (20.8) | 175 (72.9) |
| Follow Ergonomics in Practice | 2 (0.8) | 4 (1.7) | 14 (5.8) | 59 (24.6) | 161 (67.1) |
| Role of Dental Chair & Instruments | 0 (0) | 1 (0.4) | 14 (5.8) | 83 (34.6) | 142 (59.2) |
| Alternate Sitting & Standing | 1 (0.4) | 7 (2.9) | 45 (18.8) | 81 (33.8) | 106 (44.2) |
| Need for CDE | 0 (0) | 2 (0.8) | 19 (7.9) | 105 (43.8) | 114 (47.5) |

**Supplementary** **Table 3: Attitudes Toward Ergonomics in Dentistry (N = 240)**

| **ST: Ergonomic Practice Patterns (N = 240)** | | | | | |
| --- | --- | --- | --- | --- | --- |
| **Variable** | **Never** | **Rarely** | **Often** | **Very Often** | **Always** |
| Legs Position (Feet Flat) | 1 (0.4) | 13 (5.4) | 80 (33.3) | 93 (38.8) | 53 (22.1) |
| Upright Sitting with Back Support | 3 (1.3) | 44 (18.3) | 106 (44.2) | 67 (27.9) | 20 (8.3) |
| Operating Field at Elbow Level | 6 (2.5) | 40 (16.7) | 109 (45.4) | 66 (27.5) | 19 (7.9) |
| Maintain Neutral Posture | 4 (1.7) | 37 (15.4) | 109 (45.4) | 67 (27.9) | 23 (9.6) |
| Light Beam Positioning | 6 (2.5) | 30 (12.5) | 112 (46.7) | 66 (27.5) | 26 (10.8) |
| Use of Dental Loupes | 105 (43.8) | 33 (13.8) | 51 (21.3) | 30 (12.5) | 21 (8.8) |

**Supplementary Table 4: Ergonomic Practice Patterns (N = 240)**

| **ST 5: Overall Prevalence of Musculoskeletal Disorders (MSD)** | | | |
| --- | --- | --- | --- |
| **Variable** | **Category** | **Frequency (n)** | **Percentage (%)** |
| **Prevalence of MSD** | No | 60 | 25.0 |
|  | Yes | 180 | 75.0 |

**Supplementary Table 5: Overall Prevalence of Musculoskeletal Disorders (MSD)**

| **ST 6 Prevalence, Impact, Healthcare Seeking, and Work Limitation of Musculoskeletal Symptoms** | | | | |
| --- | --- | --- | --- | --- |
| **Body Region** | **12-Month Symptoms** | **Activity Limitation (12-Month)** | **Physician Visit (last 7 days)** | **Work Impact (last 7 days)** |
| Neck | 123 (51.2) | 38 (15.8) | 18 (7.5) | 67 (27.9) |
| Shoulders | 91 (37.9) | 33 (13.8) | 16 (6.7) | 51 (21.3) |
| Upper Back | 80 (33.3) | 33 (13.8) | 18 (7.5) | 44 (18.3) |
| Elbows | 35 (14.6) | 18 (7.5) | 9 (3.8) | 17 (7.1) |
| Wrists/Hands | 84 (35.0) | 28 (11.7) | 16 (6.7) | 42 (17.5) |
| Lower Back | 130 (54.2) | 52 (21.7) | 22 (9.2) | 71 (29.6) |
| Hips/Thighs | 33 (13.8) | 16 (6.7) | 8 (3.3) | 19 (7.9) |
| Knees | 48 (20.0) | 23 (9.6) | 13 (5.4) | 28 (11.7) |
| Ankles/Feet | 44 (18.3) | 25 (10.4) | 11 (4.6) | 24 (10.0) |

**Supplementary Table 6: Prevalence, Impact, Healthcare Seeking, and Work Limitation of Musculoskeletal Symptoms**

| **ST7. Logistic Regression Model Diagnostics** | |
| --- | --- |
| **Diagnostic** | **Value** |
| -2 Log Likelihood | 83.859 |
| Likelihood Ratio χ² | 67.433 |
| Degrees of freedom | 11 |
| Model p-value | <0.001 |
| Pearson Goodness-of-fit χ² | 49.261 |
| Pearson df | 81 |
| Pearson p-value | 0.998 |
| Deviance χ² | 43.457 |
| Deviance df | 81 |
| Deviance p-value | 1.000 |
| Cox & Snell R² | 0.245 |
| Nagelkerke R² | 0.363 |
| McFadden R² | 0.250 |

**Supplementary Table S7. Logistic Regression Model Diagnostics** Model fit was evaluated using likelihood ratio, Pearson goodness-of-fit, Deviance goodness-of-fit, and pseudo-R² statistics. Non-significant Pearson and Deviance goodness-of-fit tests indicate adequate model fit.
